# Astaxanthin-folic acid combined treatment potentiates neuronal regeneration and functional recovery after brachial plexus avulsion and reimplantation

**DOI:** 10.3389/fnins.2022.923750

**Published:** 2022-10-10

**Authors:** Chao Huang, Zehui Li, Wenrui Qu, Wenlai Guo

**Affiliations:** Department of Hand Surgery, The Second Hospital of Jilin University, Changchun, China

**Keywords:** brachial plexus avulsion, reimplantation, oxidative stress, inflammatory response, astaxanthin, folic acid

## Abstract

Brachial plexus avulsion (BPA), which commonly occurs in neonatal birth injuries and car accidents, severely disrupts spinal cord segments and nerve roots. Avulsion is usually located in the transitional zone at the junction of spinal nerve roots and starting point of the spinal cord, which places heavy disability burdens on patients due to sensory and motor function loss in the innervated areas. Primary mechanical injuries and secondary pathogenesis, such as inflammatory infiltration and oxidative stress, lead to inefficient management and poor prognosis. Astaxanthin (AST) has a strong ability to bleach singlet oxygen and capture free radicals, quench singlet oxygen and trap free radicals, and folic acid (FC) can effectively inhibit the inflammatory response. This study aimed to investigate the therapeutic effects of AST and FC on BPA. The 24 h after BPA was considered the acute phase of the injury, and the combination of AST and FC had the best therapeutic effect due to the synergistic effect of AST’s antioxidant and FC’s anti-inflammatory properties. At 6 weeks after BPA, AST-FC promoted the recovery of biceps motor functions, increased myofiber diameter, enlarged the amplitude of musculocutaneous nerve-biceps compound action potential, and improved Terzis grooming test (TGT) scores. Meanwhile, more functional ventral horn motor neurons in the spinal cord were maintained. In conclusion, AST-FC combined therapy has a potential role in the clinical management of BPA since it can effectively alleviate oxidative stress and the inflammatory response in the acute phase of BPA, increase the survival rate of neurons, and promote neuronal regeneration and recovery of motor functions in the late stage of BPA.

## Introduction

Brachial plexus avulsion (BPA) is one of the most severe injuries caused by preganglionic traction of the nerve roots from the spinal cord, causing huge psychological and financial burdens to patients (Wang et al., 2015). Violent injuries may cause hemorrhage and edema of the involved neuronal tissues, leading to secondary damage, including oxidative stress, inflammatory infiltration, and blood-spinal cord barrier (BSCB) damage. Oxidative stress and the inflammatory response are the primary mechanisms in secondary neuronal death (Ham and Leipzig, 2018). Reactive oxidative species (ROS) are generated around the primary injured site and lead to an oxidative stress response, resulting in the degradation of lipids, proteins, and DNA in targeted neurons (Bains and Hall, 2012; Wang et al., 2017). Moreover, BPA can cause activation of microglial cells and release of IL-6, which together with other upregulated proinflammatory cytokines, such as TNF-α and IL-1β, recruits large amounts of inflammatory cells to migrate toward the injured site, interacting to produce a series of cascade responses that peak 24–72 h postinjury (Prieto, 2008; Hayta and Elden, 2018). Therefore, early effective anti-inflammatory and antioxidative interventions are significant to alleviate secondary damage and promote neuronal regeneration and functional recovery.

Astaxanthin (AST) is a xanthophyll carotenoid widely found in algae and aquatic animals and has intense antioxidant activity as a scavenger for free radical, reactive oxygen, and nitrogen species (Krinsky, 1989; Higuera-Ciapara et al., 2006; Milani et al., 2017). Unlike other carotenoids, it contains two other oxygen-containing groups in each ring structure, enhancing its antioxidant properties, and studies have shown that AST is more than ten times more capable of quenching singlet oxygen and trapping free radicals than β-carotene (Krinsky, 1989; Fassett and Coombes, 2009). AST is one of the most abundant carotenoids in the diet, has been used as a therapeutic agent for various diseases *in vivo* and *in vitro* without showing any side effects or toxicity (Higuera-Ciapara et al., 2006; Zhang et al., 2014). Fakhri et al. showed that AST could significantly preserve the number of myelinated white matter and motor neurons after spinal cord injury. Its effects were mediated by a reduction in activated ERK signaling pathways while an increase in activated AKT signaling pathways (Fakhri et al., 2019). Han et al. used AST to treat Alzheimer’s disease (AD) rats. They showed that AST could significantly increase superoxide dismutase (SOD) and reduce the malondialdehyde (MDA) and lactate dehydrogenase (LDH) content in the hippocampal tissue, thereby reducing oxidative stress in AD rats and promoting long-term functional recovery (Han et al., 2018). Wu et al. found that AST could activate nuclear factor erythroid-related factor 2 and the antioxidant responsive element (Nrf2-ARE) pathway in subarachnoid hemorrhage model rats, resulting in upregulating cortical endogenous antioxidant levels, reducing oxidative damage, brain edema, and disruption of the blood-brain barrier (BBB), inhibiting apoptosis, as well as improving neurological function and ameliorating brain injury (Wu et al., 2014).

Folic acid (FC) is a water-soluble B vitamin that can be reduced to tetrahydrofolate *in vivo* in response to folate reductase and is involved in the synthesis and conversion of purines, pyrimidines, and amino acids (Heseker, 2011; Ohrvik and Witthoft, 2011; Baltrusch, 2021). Studies have shown that FC is associated with the hematopoietic system and the growth and development of the nervous system, where it plays a crucial role in the proliferation and differentiation of neural stem cells (Amarin and Obeidat, 2010; Liu et al., 2010; Stewart et al., 2019). Its physiological mechanisms are gradually being discovered with the increasing use of FC. Among them, the attenuation of the inflammatory response is a potential mechanism (Wang et al., 2005). Kolb and Petrie (2013) showed that the expression of the inflammatory mediators IL-1β, IL-6, TNF-α, and MCP1 was increased 2- to 3-fold in the presence of folate deficiency in RAW264.7 cells (Kolb and Petrie, 2013). Cianciulli et al. showed that FC significantly attenuated the release of proinflammatory mediators in LPS-activated microglia, blocked the nuclear factor kappa B (NF-κB) pathway, and promoted the release of anti-inflammatory mediators by overregulating IL-10-dependent SOCS protein expression through the p38 pathway, thereby inhibiting the production of proinflammatory factors and exerting anti-inflammatory effects (Cianciulli et al., 2016). Zhang and Shen showed that FC significantly reduced the expression of inflammatory proteins in neuronal cells, prevented Ca^2+^ overload under hypoxic conditions, significantly upregulated Notch1 mRNA and protein expression, and had a significant protective effect on neuronal cells. Combined treatment with FC and adult neural stem cells significantly reduced the inflammatory response of spinal cord tissue, restored damaged nerve cells, and significantly improved neurological function (Zhang and Shen, 2015).

There are currently no studies that apply AST or FC to the treatment of BPA. Therefore, this study investigated the efficacy of AST and FC as antioxidant and anti-inflammatory intervention drugs in promoting neural regeneration and functional recovery in BPA rats.

## Materials and methods

### Materials

AST and FC were purchased from Solarbio Co., Beijing and Sigma–Aldrich (USA), respectively. Cook-level olive oil was ordered from Córdoba (Spain). Isoflurane (2.5%) was purchased from Keyue Huacheng Co., Beijing. Absorbable gelatin sponges were obtained from Jinling Pharmaceutical Company, Nanjing. IL-6 ELISA kits were purchased from Mabtech Company (Sweden). We obtained Fluoro-Gold (FG) from Fluorochorome (USA) and anti-goat rabbit 488 antibodies from Abcam (USA). Anti-rat goat ChAT antibodies were ordered from Millipore (Germany).

### Grouping of animals

Seventy-two adult SPF female SD rats, 10 weeks old and weighing 250 g, were provided by the Laboratory Animal Center of Jilin University and were cultivated in a standardized animal room at a constant temperature of 22°C. One single breeder controlled all the cages. Pellet feed and water were routinely provided without limitation. Before making the BPA model, the motor function of the forelimb in all rats was examined to exclude any original abnormalities, and then these qualified animals were divided into four groups (*n* = 18): control group, AST group, FC group, and AST + FC group. The therapeutic dose of AST was 75 mg/kg. AST was diluted in olive oil (1 mL/kg) and given twice a day from postsurgical 3 h to 2 weeks *via* oral intragastric administration (Wang et al., 2019). FC was intraperitoneally injected twice a day from 3 days preoperatively to 2 weeks postoperatively at 80 μg/kg (Iskandar et al., 2004). Administrative details were shown in Table 1.

**TABLE 1:** Group and treatment.

Treatment	Control group	AST group	FC group	AST + FC group
Oral intragastric administration	1 mL/kg olive oil	75 mg/kg AST	1 mL/kg olive oil	75 mg/kg AST
Intraperitoneal injection	Normal saline	Normal saline	80 μg/kg FC	80 μg/kg FC

AST, astaxanthin; FC, folic acid.

### Brachial plexus avulsion model construction

**FIGURE 1:**
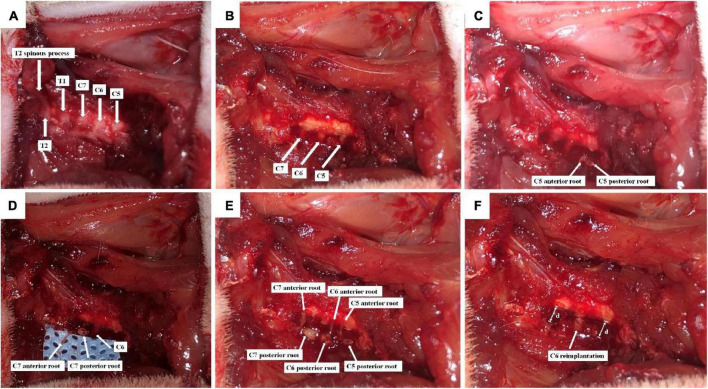
Schematic diagram of C6 nerve root reimplantation after BPA. **(A)** C5-T2 laminae were exposed marked by T2 spinous process; **(B)** the right C4–C7 laminae were excised, and C5–C7 segments together with their dorsal roots were exposed; **(C)** C5 dorsal root was cut, and the ventral root was torn off; **(D)** C7 dorsal root was cut, and the ventral root was torn off; **(E)** C6 dorsal root was cut, and the ventral root was torn off. C5–C7 were placed on the surface of the spinal cord; **(F)** 5–7 mm (d) nerve tissues were excised from the dorsal and ventral roots of C5 and C7, so that distal nerve roots were retracted. Then, C6 dorsal and ventral roots were reimplanted.

After adaptation for 1 week, all rats were anesthetized with 2.5% isoflurane, and the BPA model was established by the same person based on the GU’s method (Figures 1A–F; Gu et al., 2004). Briefly, the backs of rats were depilated, and the rat was fixed on a sterile operating table in a prone position. After routine disinfection, a posterior median incision was made from the occipital region to the suprascapular angle, approximately 3 cm long, using the second thoracic spine (T2) as a bony landmark (Figure 1A), and muscles on the surface of the right vertebral body were separated layer by layer, and the lamina and spinous processes from the fourth cervical spine (C4) to the T2 were exposed. Under a light microscope, the right C4–C7 vertebral plates were removed using a rongeur, and the right C5–C7 spinal nerve roots were carefully isolated (Figure 1B). The dorsal roots of C5–C7 were cut, and the ventral roots were torn off with a self-made glass hook (Figures 1C–E). Then, a segment of 5–7 mm nerve tissue between the peripheral nerve and the spinal cord was excised from C5 and C7 dorsal and ventral roots to avoid their regeneration. Finally, C6 dorsal and ventral roots were reimplanted to the spinal cord (Figure 1F), and the muscles and skin were sutured after sufficient hemostasis.

Intramuscular administration of 50 mg/kg cefazolin sodium (Zhongnuo Pharmaceutical Company, Shijiazhuang) was performed twice a day for 3 days postoperatively to prevent postsurgical infections. The Terzis grooming test (TGT) was performed and scored on the first and second days postsurgery (Inciong et al., 2000; Hao et al., 2018). The detailed scores of the TGT were shown in Table 2. Scoring 0 indicated a successful BPA model for further experiments.

**TABLE 2:** Terzis grooming test.

Grade	Performance in the grooming test	Score
0	Rats without any reaction.	0
1	Rats can elbow but cannot touch their nose.	1
2	Rats can elbow and touch their nose.	2
3	Rats can elbow to the position below their eyes.	3
4	Rats can elbow to their eyes.	4
5	Rats can elbow to their ear or the back position of ears.	5

### Measurement of reactive oxidative species levels during the acute phase in injured spinal cord tissues *via* the immunofluorescence method

Rats were randomly selected in each group 24 h postsurgery and intraperitoneally injected with 1 mg/mL dihydroethidium (DHE) (10 mg/kg) (Zhang et al., 2016). Animals were then anesthetized after 4 h with 2.5% isoflurane. Cardiac perfusion *via* 0.9% normal saline and 4% paraformaldehyde was successively performed. C5–C7 spinal cord segments were dissected and dehydrated with 30% sucrose solution. Slides (10 μm) were cross-sectioned using a cryostat, and an inverted fluorescence microscope took their photos under a 570 nm excitation light source. Five regions of interest measuring 100 × 100 μm in size were randomly selected in the ventral horns, and the mean fluorescence density was quantified and analyzed using Image-Pro Plus software. The results were interpreted as relative fluorescence intensities.

### Measurement of IL-6 quantification during the acute phase in injured spinal cord tissues *via* spectrophotometer

Twenty-four hours postsurgery, 3 rats were randomly selected from each group, anesthetized, and perfused with 0.9% saline. Low-temperature surroundings were achieved by placing fresh ice around the bench. C5–C7 spinal cord tissues were dissected, mixed with homogenizing buffer (pH = 6.0, PBS 50 mM, 0.5% cetyl amine bromide), and homogenized for 10 min. The above homogenates were ultrasonicated for 10 s and freeze-thawed 3 times between a −80°C freezer and 37°C incubator. Then, the samples were centrifuged at 14,000 rpm/min for 20 min (4°C). Supernatants were collected, and IL-6 contents were then measured by ELISA kits (Mabtech, Sweden).

### Behavioral assays

Two- to six-week postsurgery, two researchers blinded to previous BPA model constructs assessed the locomotory functions of biceps on the injured limb once a week by the TGT scoring method. The third person would become involved and independently score the behavioral performance if disagreement appeared.

### Measurement of C6 ventral motor neuron survival rate *via* immunofluorescence method

Six weeks postsurgery, 3 rats per group were randomly selected. Spinal cord tissues were collected using the same method as described in section Measurement of ROS levels via the immunofluorescence (IF) method and used for ChAT staining. The biceps were also dissected for further hematoxylin-eosin (H&E) staining. One out of every four sections was incubated in anti-ChAT primary antibody (1:100; Millipore) at 4°C overnight and then in anti-goat rabbit 488 antibody (Abcam) at room temperature for 1.5 h. ChAT-positive (+) C6 ventral horn motor neurons with normal morphology were counted under a fluorescence microscope, and the ratios of qualified neurons between the injured side and contralateral normal side were calculated.

### Measurement of C6 ventral horn motor neurons *via* musculocutaneous nerve fluoro-gold retrograde labeling

Three rats per group were randomly selected 6 weeks postsurgery, and after anaesthetization, transversely incised under the right clavicle to cut off the pectoralis major and minor. Then, the musculocutaneous nerve was exposed and mobilized. The point 5 mm proximal to the musculocutaneous nerve-muscle junction was chosen as the entry site for the 10 μm glass needle, and 1 μL FG was infused into the musculocutaneous nerve *via* a microinjection pump. The tip of the needle remained inside the tissues for at least 10 s until FG was fully absorbed. Cefazoline sodium was routinely administered after microinjection to prevent any potential infections (as described before). C5-C8 spinal cord tissues were harvested 3 days later using the same protocol as described before. Slides (25 μm) were longitudinally sectioned using a cryostat. One out of every four sections was observed under a fluorescence microscope, and the numbers of FG-labeled neurons were counted.

### Hematoxylin-eosin staining of injured biceps myofibers

Biceps samples were harvested and longitudinally cut into 14 μm slides with a cryostat. One out of every four sections was obtained for H&E staining. One hundred myofibers per biceps sample were randomly selected and photographed under a light microscope. Myofiber diameters were measured by ImageJ software, and mean values were then calculated.

### Electrophysiological examination of biceps-musculocutaneous nerve compound action potentials

Electromyography (EMG) recording was performed in the selected 3 rats per group 6 weeks postsurgery. After anaesthetization, the right chest and anterior limbs surfaces were shaved and disinfected. The pectoralis major and minor were separated to expose the biceps and musculocutaneous nerve underneath. A pair of stimulus electrodes hooked the musculocutaneous nerve, and another pair of recording electrodes were directly inserted into the biceps at a depth of approximately 2 mm. The distance between the two electrodes was approximately 5 mm, and the stimulating voltage was 1.2 mV. At least three different sites were tested three times separately on each muscle unit. The amplitude of the evoked potential was recorded at each site. The mean amplitude was then calculated for further statistical analysis and comparison.

### Statistical analysis

Figures were generated *via* GraphPad Prism 7 (Graph Pad Software, Inc., CA, USA). One-way ANOVA (analysis of variants) was conducted by SPSS 22. The results are shown as the mean ± SD. For pairwise comparisons, Student’s *t*-test was used. Here, we considered *p* < 0.05 to be statistically significant.

## Results

### Astaxanthin/folic acid treatment inhibited oxidative stress and the inflammatory response in the acute phase of brachial plexus avulsion

**FIGURE 2:**
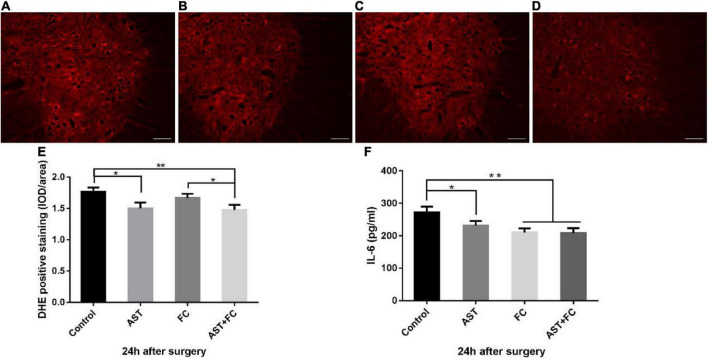
Fluorescence intensity of DHE and IL-6 levels at 24 h after BPA in the control, AST, FC, and AST + FC groups. **(A–D)** DHE fluorescence intensity in the anterior horn of the spinal cord at 24 h after BPA and represent the control, AST, FC, and AST + FC groups, respectively (indicated by red areas, bar = 100 μm). **(E)** Statistical analysis and comparison of average DHE fluorescence intensities in spinal cord ventral horns in control, AST, FC, and AST + FC groups at 24 h after BPA (*n* = 3, *p < 0.05, **p < 0.01). **(F)** Statistical analysis and comparison of average IL-6 levels in C5–C7 spinal cord tissues in control, AST, FC, and AST + FC groups at 24 h after BPA (*n* = 3, *p < 0.05, **p < 0.01).

Compared to the control group (1.76), ROS levels in treated individuals were significantly lowered at 24 h after BPA, which was interpreted by the decrease in DHE fluorescence intensity, with average DHE fluorescence intensities of 1.50, 1.67, and 1.48 for the AST, FC, and AST + FC groups, respectively (Figures 2A–D). Among them, a statistically significant decrease only appeared in the AST + FC group and AST group compared with the control group (Figure 2E, *p < 0.05, ^**^p < 0.01).

On the other hand, IL-6 levels in C5-C7 segment spinal cord tissues at 24 h after BPA were all significantly lower in the AST, FC, and AST + FC groups than in the control group, with IL-6 levels of 231.6, 210.4, 208.8, and 272.3 pg/mL for the AST, FC, AST + FC, and control groups, respectively (Figure 2F, *p < 0.05, ^**^p < 0.01).

### Astaxanthin/folic acid treatment promoted recovery of biceps motor function after brachial plexus avulsion

**FIGURE 3:**
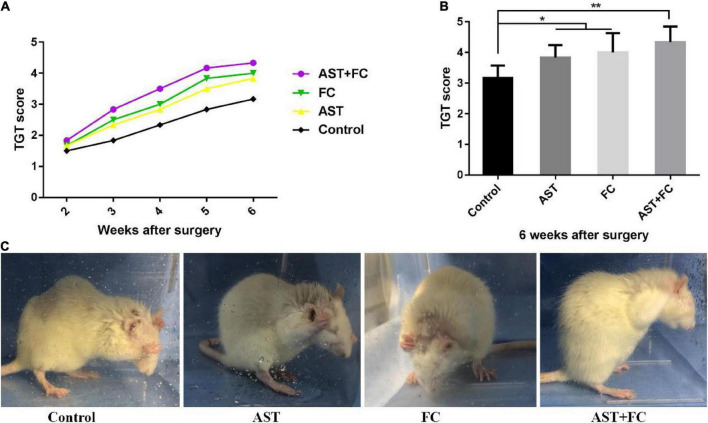
The postsurgical motor function of the involved limbs in the control, AST, FC, and AST + FC groups. **(A)** Each week’s TGT scores during the 2nd to 6th week in control, AST, FC, and AST + FC groups after BPA (*n* = 6); **(B)** average TGT scores in control, AST, FC, and AST + FC groups at the end of the 6th week after BPA (*n* = 6, *p < 0.05, **p < 0.01); **(C)** images of the gross motor function of the involved forelimb in control, AST, FC, and AST + FC groups at the 6th week after BPA.

At 2–6 weeks after BPA, the motor function of the rats from the control group progressively recovered over time, as indicated by the steady rise tendency in their TGT score curve. Up to the 6th week, the TGT score had turned to 3.17. In the AST and FC groups, TGT scores were higher than those of the control group but lower than those of the AST + FC group during these 4 weeks. These results concluded that AST and FC both promoted the recovery of biceps motor function post-BPA. Regarding TGT scores, the AST group demonstrated steady growth at 2–6 weeks postsurgery, whereas the FC group showed steady growth from the 2nd to 4th week, followed by a faster tendency from the 4th to 5th week and a slower tendency from the 5th to 6th week. However, in general, TGT scores in the FC group were consistently higher than those in the AST group (Figure 3A). Finally, at the 6th week postsurgery, the TGT scores of the AST (3.83) and FC (4.00) groups were both significantly higher than that of the control group (3.17) (Figures 3B,C, *p < 0.05, ^**^p < 0.01). Moreover, TGT scores in the AST + FC group were maintained at the highest level during the post-BPA period with constant steady growth, indicating better motor function recovery. Notably, the TGT score in the AST + FC group reached 4.33 in the 6th week, with a statistically significant difference compared to the control group (Figures 3A–C, *p < 0.05, ^**^p < 0.01).

### Astaxanthin/folic acid treatment improved the survival rate of ventral horn motor neurons on the injured side

**FIGURE 4:**
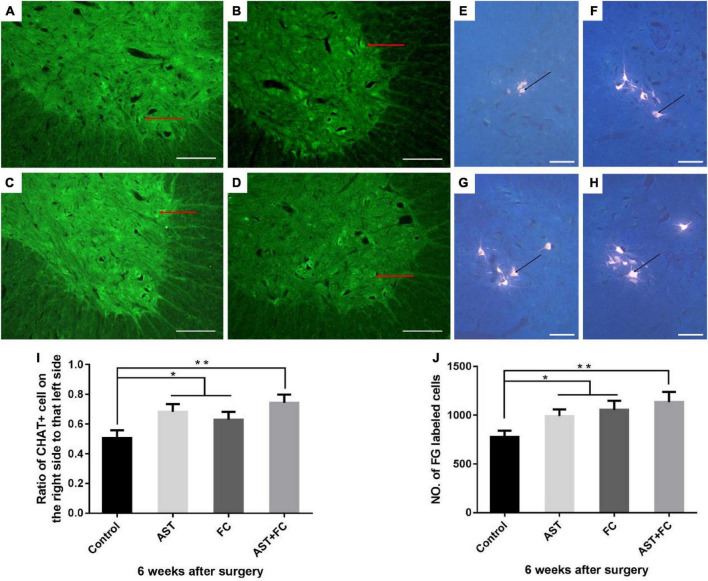
Quantification of viable ventral motor neuron counts and survival rates, and the number of FG-labeled ventral horn motor neurons on the injured side in control, AST, FC, and AST + FC groups at 6 weeks after BPA. **(A–D)** The number and morphology of ventral horn motor neurons on the injured side at 6 weeks after BPA and represent the control, AST, FC, and AST + FC groups, respectively (bar = 100 um); **(E–H)** C5–C8 FG-labeled ventral horn motor neurons in control, AST, FC, and AST + FC groups at 6 weeks after BPA and represent the control, AST, FC, and AST + FC groups, respectively (the black dots indicate ventral horn motor neurons, bar = 50 μm). **(I)** Ratios between the percentage of viable ventral horn motor neurons on the injured side and that on the healthy side (*n* = 3, *p < 0.05, **p < 0.01). **(J)** Statistical comparison of the number of FG-labeled motor neurons in each group (*n* = 3, *p < 0.05, **p < 0.01).

At 6 weeks after BPA, the cell bodies of ventral horn motor neurons from the AST, FC, and AST + FC groups all enlarged, and the numbers of ventral horn motor neurons alive increased compared to the control group (Figures 4A–D). The ratios of viable ventral horn motor neurons on the injured side vs. the healthy side were 50.67, 68.33, 63.00, and 72.33% in the control, AST, FC, and AST + FC groups, respectively (Figure 4I). Among them, the ratios of the AST, FC, and AST + FC groups were significantly higher than that of the control group (Figure 4I, *p < 0.05, ^**^p < 0.01). Apart from the increasing number of ChAT (+) ventral horn motor neurons at the C6 level in the AST, FC, and AST + FC groups, there were other morphological differences from the control group. The cell bodies of neurons in the AST, FC, and AST + FC groups were remarkably enlarged compared with those in the control group, with axons and synapses visible in certain regions.

### Astaxanthin/folic acid treatment increased the number of fluoro-gold-labeled functional ventral horn motor neurons on the injured side

At 6 weeks after BPA, the cell bodies of the FG-labeled neurons in the AST, FC, and AST + FC groups were greatly enlarged compared with those in the control group, with a clearer view of axonal and synaptic growth (Figures 4E–H). Meanwhile, the average number of FG-labeled C5–C8 ventral horn motor neurons significantly increased in the AST group (990.7), FC group (1054.0), and AST + FC group (1134.0) compared with the control group (777.3) (Figure 4J, *p < 0.05, ^**^p < 0.01).

### Astaxanthin/folic acid treatment accelerated the growth of the injured biceps myofibers

**FIGURE 5:**
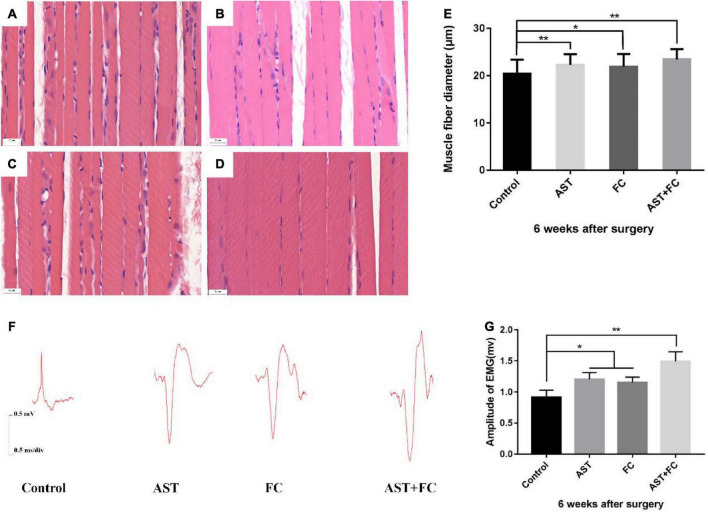
Diameters of biceps myofibers and the amplitude of compound action potential of musculocutaneous nerve-biceps on the injured side at 6 weeks after BPA. **(A–D)** Diameters of biceps myofibers from the control, AST, FC, and AST + FC groups (*n* = 3, bar = 20 μm). **(E)** Statistical comparison of the diameters from the above 4 groups (*p < 0.05, **p < 0.01). **(F)** Compound action potentials of musculocutaneous nerve-biceps in control, AST, FC, and AST + FC groups at 6 weeks after BPA. **(G)** Statistical analysis of amplitudes of compound action potentials from the 4 groups (*n* = 3, *p < 0.05, **p < 0.01).

H&E staining of biceps myofibers on the injured limb at 6th week after BPA showed that the average myofiber diameter in the AST + FC group (23.44 μm) was higher than that in the control group (20.44 μm), followed by the AST group (22.30 μm) and FC group (21.90 μm) (Figures 5A–E, *p < 0.05, ^**^p < 0.01).

### Astaxanthin/folic acid treatment increases the amplitude of the compound action potential of the musculocutaneous nerve-biceps on the injured side

According to the compound action potential results of the musculocutaneous nerve-biceps on the injured limb at 6th week after BPA, the average amplitude was 1.203 and 1.153 mV in the AST and FC groups, respectively, which were both higher than that in the control group (0.916 mV). The maximal amplitude appeared in the AST + FC group, which was 1.493 mV with a statistically significant difference compared to the control group (Figures 5F,G, *p < 0.05, ^**^p < 0.01).

## Discussion

In 70% of severe strains of the brachial plexus, it is accompanied by avulsion of the nerve roots, causing permanent separation of the spinal cord from the peripheral muscles, resulting in irreversible death of ventral horn motor neurons due to loss of nutrition and accumulation of toxic metabolites, preventing nerve regeneration and causing permanent loss of motor function (Carlstedt and Havton, 2012). BPA is a preganglionic nerve injury and is generally treated with nerve grafts or functional muscle graft reconstruction, which carries the risk of compensating for normal nerve tissue and requiring secondary surgery (Richardson et al., 1982). *In situ* reimplantation of the avulsed nerve is gaining increasing attention because of its simplicity and minimal side effects of injury (Carlstedt et al., 2000; Gu et al., 2004). However, *in situ* reimplantation alone does not provide satisfactory postoperative functional recovery (Huang et al., 2021).

Studies have shown that BPI is usually accompanied by significant neuroinflammation, oxidative stress, and BSCB disruption (Liu et al., 2020). In these secondary injuries, oxidative stress and inflammatory responses at the injury site are the most important processes leading to the massive death of motor neurons (Oliveira and Langone, 2000; Oyinbo, 2011). The avulsion injury results in excessive production of ROS, including O_2_^•–^, H_2_O_2_, and ^•^OH, which disrupt the body’s antioxidant capacity and cause oxidative damage at the injury site, inducing lipid peroxidation and protein and DNA damage (Bullon et al., 2015). Excess ROS are associated with neurodegenerative diseases and are also considered to be a major disruptive factor in nerve damage (Halliwell, 2006; Hall et al., 2016). In addition, the abnormally activated inflammatory response is also an important mechanism mediating nerve cell damage. As the primary immune cells of the nervous system, microglia are the primary source of the neuroinflammatory response (Liu et al., 2020). Under the stimulation of pathological factors, microglia are significantly activated and aggregated within the lesion to remove debris generated by damaged tissue and repair damaged neurons. However, when overactivated, microglia produce excessive proinflammatory cytokines such as IL-1β, IL-6, and TNF-α. These cytokines directly cause neuronal damage and inhibit axonal and dendritic regeneration (Prieto, 2008). Thus, antioxidant and anti-inflammatory therapies may effectively decrease neuronal death and create the foundation for nerve regeneration.

Due to the multiple double bonds in the polyene chain, AST has strong antioxidant properties and can absorb free radicals during oxidative stress. Previous studies have shown that AST can enhance the activities of SOD and catalase (CAT) and promote the level of reduced glutathione (GSH). It also reduces lipid peroxidation by lowering MDA levels at the injury site (Grimmig et al., 2017; Hong et al., 2020). Moreover, the specific structure of AST allows it to cross the BBB for easy use in neurological disorders. Therefore, the application of AST in BPA treatment is promising (Grimmig et al., 2017; Castelli et al., 2020). FC can regulate the inflammatory response of microglia by regulating various signaling pathways, and it has been widely reported. FC reduced the production of proinflammatory cytokines, including TNF-α, IL-1, and NO, in microglia and increased the production of SOCS1 and SOCS3 by upregulating p38 MAPK phosphorylation and downregulating p-IB and JNK phosphorylation. In contrast, the expression of the anti-inflammatory cytokine IL-10 increased, realizing the regulation of the inflammatory response of microglia, avoiding cells being damaged by the inflammatory response (Cianciulli et al., 2016). Moreover, Cheng et al. found that the microglial immune response mediated by the Notch1/NF-κB p65 pathway is the molecular mechanism by which FC deficiency aggravated neuronal cell death and microglial activation in the hippocampus after cerebral ischemia/reperfusion (Cheng et al., 2019).

This study is the first to use AST and FC as antioxidant and anti-inflammatory drugs to treat BPA injury. We chose the dorsal entrance to avulse the right C5–C7 nerve roots, which innervated the ipsilateral musculocutaneous nerve and biceps. A 5–7 mm gap was left after cutting the proximal ends of avulsed C5 and C7 nerve roots to prevent further regeneration of the corresponding nerves. The only autografted and reimplanted nerve root was C6, generating a circuit in which one single nerve root innervated only one single peripheral nerve or muscle effector. This single-to-single circuit diminished other potential interruptive biases to potentiate the observation of AST-FC treatment efficiency. At 24 h after reimplantation, the AST group and FC group showed a significant treatment efficiency: the AST group behaved better against oxidative stress, while FC had a more substantial anti-inflammatory effect. Furthermore, the combination of AST and FC showed a synergistic effect, which efficiently lowered post-BPA oxidative stress and inflammatory infiltration in the acute phase and protected neurons.

ChAT primarily catalyzes acetylcholine synthesis, the marker enzyme of cholinergic neurons, in response to some physiological reactions. It is produced intracellularly, stored in nerve terminals *via* axonal transport, and modulates the motor function of target organs (muscle) by regulating acetylcholine synthesis. Detection of ChAT in peripheral nerves or muscles can somehow reflect the regeneration level of peripheral nerves after nerve root replantation. Therefore, ChAT immunostaining has already been applied to measure ventral horn motor neurons (Blits et al., 2004). Rende et al. found that neuronal cells did not express ChAT within 7 days after nerve avulsion injury, while the expression of ChAT was upregulated until 30 days after injury (Rende et al., 1995). Our study used IHC to detect the expression of ChAT in motor neurons in the ventral horn of the spinal cord. ChAT (+) neurons can indirectly reflect the changes in the number of ventral horn motor neurons in the C6 segment that were replanted after BPA injury.

Furthermore, retrograde injection of FG can also quantify the regenerative level of reimplanted nerves. Novikova et al. reported the specificity of FG retrograde labeling for observing neurons. FG only labeled functional, healthy motor neurons that have established contact with distal nerve fibers instead of dead or abnormal neurons (Novikova et al., 1997). FG labeled the reimplanted neurons from the corresponding section level through retrograde transportation from the injured peripheral nerves. FG-labeled neurons in the reimplanted site remarked that the axonal connection between spinal cord neurons and peripheral nerves was regenerated (Gallent and Steward, 2018). In our study, the AST group, FC group, and AST-FC group showed a remarkable increase in ChAT (+) and FG-labeled motor neurons compared with the control group. Meanwhile, cell bodies were also enlarged after treatment, and the numbers of axons and synapses also increased, which was more evident in the AST + FC group. Therefore, early anti-inflammation and antioxidation treatment after BPA could better protect neuronal survival and promote axonal regeneration.

The final goal for BPA treatment is to restore the motor function of the involved limb as much as possible. The biceps are the major effector of the musculocutaneous nerve, the lateral branch of the brachial plexus (Duijnisveld et al., 2017). Neuronal signals are disconnected between the musculocutaneous nerve and CNS after nerve root avulsion, and progressive myofiber atrophy and fibrosis successively appear due to muscular denervation. If the innervation signals fail to be reconstructed in time, even if a nerve signal arrives later, it will not establish effective motor units to meet the needs of functional recovery, resulting in loss of muscle motor function. Previous studies revealed that the reimplantation of avulsed nerve roots also resulted in the implantation of Schwann cells, which are critically essential for peripheral nerves. After axonal transection, Schwann cells grow, produce neurotrophic factors, and synthesize laminin in the extracellular matrix. Meanwhile, they also produced various cell adhesion molecules to stimulate axonal regeneration, protected the injured motor neurons, and further recovered innervation of distal effector (muscle) (Acheson et al., 1991; Martini, 1994).

In this study, we took the biceps myofibers on the injured limb 6 weeks after BPA, conducted HE staining, and measured their diameters. An increase in muscle diameter indicates recovery of motor function. Interestingly, the AST + FC group showed the most significantly enlarged myofiber diameter, indicating the best recovery of motor function due to effective anti-inflammatory and antioxidative treatment. TGT scores could also verify this conclusion at 2–6 weeks after BPA. The TGT scores of the AST group, FC group, and AST + FC group were higher than those of the control group at 2–6 weeks after BPA, showing the macroscopic effect of the treatment on the kinematic score. It is noteworthy that the FC group demonstrated higher scores than the AST group from the beginning, probably because FC cannot be synthesized *in vivo*. Exogenous FC can be converted to THF as the primary vector of one-carbon units to synthesize purines, pyrimidines, and amino acids, providing the primary raw materials for postsurgical neuronal recovery (Heseker, 2011). According to the TGT results, the motor function of the affected limb could gradually recover but could still not return to its original level, which was closely related to the irreversible death of motor neurons after BPA, in agreement with the view of Shin et al. (Shin et al., 2005). Electrophysiological examination is an important method to assess the recovery level of nerve-muscle function. In this study, amplitudes of the musculocutaneous nerve-biceps compound motor potential of the AST group, FC group, and AST + FC group were higher than that of the control group, indicating better recovery of neuromuscular motor function after treatment.

## Conclusion

The combined application of AST and FC in our BPA model effectively decreased oxidative stress and the inflammatory response during the acute phase, preserved more functional ventral horn motor neurons in the anterior horn, and promoted nerve regeneration at the injury site and recovery of motor function of the involved limb, showing a significant therapeutic effect.

## Data availability statement

The original contributions presented in this study are included in the article/supplementary material, further inquiries can be directed to the corresponding author.

## Ethics statement

This study was approved by the Ethical Committee of experimental animals at Jilin University. All the animal-involved procedures were based on the “Guide for the Care and Use of Laboratory Animals” National Research Council (US) (2011). Essential preventions were conducted to minimize the suffering of laboratory animals, and the total number of animals used in our study was strictly controlled.

## Author contributions

CH and ZL designed the study, performed the literature review, extracted the data, and analyzed the pooled data. CH drew the figures and organized the tables. WQ and WG provided critical comments and revised the manuscript. All authors read and approved the final manuscript.
